# The Human Capital of Knowledge Brokers: An analysis of attributes, capacities and skills of academic teaching and research faculty at Kenyan schools of public health

**DOI:** 10.1186/s12961-016-0133-0

**Published:** 2016-08-02

**Authors:** Nasreen Jessani, Caitlin Kennedy, Sara Bennett

**Affiliations:** Department of International Health, Johns Hopkins University Bloomberg School of Public Health, 615 N. Wolfe St, Baltimore, MD 21205 United States of America

**Keywords:** Kenya, Knowledge broker, Attribute, Capacity, Skill, Evidence-to-policy, Qualitative, Schools of public health

## Abstract

**Background:**

Academic faculty involved in public health teaching and research serve as the link and catalyst for knowledge synthesis and exchange, enabling the flow of information resources, and nurturing relations between ‘two distinct communities’ – researchers and policymakers – who would not otherwise have the opportunity to interact. Their role and their characteristics are of particular interest, therefore, in the health research, policy and practice arena, particularly in low- and middle-income countries. We investigated the individual attributes, capacities and skills of academic faculty identified as knowledge brokers (KBs) in schools of public health (SPH) in Kenya with a view to informing organisational policies around the recruitment, retention and development of faculty KBs.

**Methods:**

During April 2013, we interviewed 12 academics and faculty leadership (including those who had previously been identified as KBs) from six SPHs in Kenya, and 11 national health policymakers with whom they interact. Data were qualitatively analyzed using inductive thematic analysis to unveil key characteristics.

**Results:**

Key characteristics of KBs fell into five categories: sociodemographics, professional competence, experiential knowledge, interactive skills and personal disposition. KBs’ reputations benefitted from their professional qualifications and content expertise. Practical knowledge in policy-relevant situations, and the related professional networks, allowed KB’s to navigate both the academic and policy arenas and also to leverage the necessary connections required for policy influence. Attributes, such as respect and a social conscience, were also important KB characteristics.

**Conclusion:**

Several changes in Kenya are likely to compel academics to engage increasingly with policymakers at an enhanced level of debate, deliberation and discussion in the future. By recognising existing KBs, supporting the emergence of potential KBs, and systematically hiring faculty with KB-specific characteristics, SPHs can enhance their collective human capital and influence on public health policy and practice. Capacity strengthening of tangible skills and recognition of less tangible personality characteristics could contribute to enhanced academic–policymaker networks. These, in turn, could contribute to the relevance of SPH research and teaching programs as well as evidence-informed public health policies.

## Background

### Knowledge brokers (KBs)

Known by a host of synonymous terms [1–6], KBs serve as the link and catalyst for knowledge synthesis and exchange between researchers and policymakers [1, 7, 8]. Their role is of particular interest in health research, policy and practice [9–12], where actors are often isolated, siloed [2, 13–17] and challenged to support and promote evidence-informed decision-making (EIDM) [18].

In health policy and systems research, several types of actors have been recognised as KBs, including science journalists [8], knowledge translation platforms [19–21], advocacy coalitions [22], and development partners and funders [23], amongst others. Emerging among these KBs are academic faculty playing a dual role: that of knowledge generator as well as of KB. These academics have been referred to as ‘hybrid’ or ‘blended’ professionals [6] who are connected to both worlds they bridge and thus occupy a ‘double peripherality’ [8, 24]. For the purpose of this paper we define them as academic KBs – faculty who are not only well connected to policymakers but also sought out by their peers for advice and knowledge on strategies to engage in EIDM.

Network theory defines social capital [25, 26] as “*the advantage created by a person’s location in a structure of relationships*” [27] and human capital [28, 29] as “*a person’s advantage in terms of personal attributes*” [27], capacities and skills that facilitate the creation and maintenance of relationships. Network theory therefore provides a platform for systematically reviewing the identification and characteristics of KBs.

Previous studies specific to academic–policymaker partnerships, and to knowledge-brokering in particular, have varied in their foci ranging from frameworks to capture the complex dynamics of interactions between actors [30, 31] and the organisational culture in which they operate [8, 32, 33], to the roles of KBs and the fluid nature of their suite of activities [34–37], to the individual skills of KBs [4, 6, 14, 23, 38, 39], their motivations [7, 40–43] and their personalities [40, 44] or qualities [45]. However, most of the studies are informed by Western experiences and none of the frameworks to-date capture the attributes, capacities and skills of academic KBs specific to low- and middle-income countries. While understanding the power dynamics, the environmental and political factors and the organisational culture are important in understanding what shapes individual actions, in this paper we focus on the sociodemographic characteristics as well as human capital of academic KBs contributing to public health EIDM in Kenya. Specifically, we seek to understand the individual attributes, capacities and skills of individuals identified as KBs in schools of public health (SPH) in Kenya with a view to informing organisational policies around the recruitment, retention and development of KBs.

### Context

At the time of this study (March–May 2013) Kenya was transitioning to a decentralised health system under Vision 2030 – the country’s development blueprint for the period 2008–2030 [46]. The national government was increasingly adopting a stewardship role while devolving management of service delivery and funding to counties [46]. Vision 2030’s emphasis on, investment in, and utilisation of scientific research to advance development caused a shift in the landscape of attitudes towards, value for, and demand on academia.

Kenya has several institutions of higher education, including universities, colleges, vocational and technical training institutes. Of Kenya’s approximately 39 universities [47], six have dedicated programs for public health research and training: University of Nairobi School of Public Health (SPHUoN), Kenyatta University School of Public Health, Kenya Methodist University (KEMU), Maseno University School of Public Health and Community Development (ESPUDEC), Moi University School of Public Health (MUSOPH), and Great Lakes University of Kisumu – Tropical Institute for Community Health (GLUK-TICH). While not all institutions were classified as SPHs within their organisations, we refer to them collectively as SPHs for the purposes of this study. We did not include other organisations engaged in research and related EIDM activities, as none were embedded in university contexts where teaching, research and service compound the dual role of generating as well as translating evidence.

### Academic KBs in Kenya

The increasing demand for transparency from civil society, the strict accountability requirements of external funders and the emerging role of KBs places Kenya in a precarious position should it fail to uphold its commitments to invest in EIDM. The importance, therefore, of generating, accessing and utilising policy-relevant health systems evidence has attracted the attention of researchers and policymakers alike and has particular implications for SPHs, and the faculty within, who are well placed to provide this evidence base. However, little is known about the characteristics of such faculty. We initially therefore sought to answer the question: Do academic KBs exist within Kenyan SPHs? [48]. Upon identifying seven KBs amongst 124 surveyed faculty, we further sought to understand faculty as well as policymaker perceptions with respect to personal attributes, skills and capacities that facilitate or hinder knowledge brokering and to explore the implications of the results on SPH hiring practices and SPH capacity strengthening endeavours.

## Methods

### Data collection

Academic KBs were initially identified using Social Network Analysis (SNA) as a heuristic device for actor identification and network explorations [49]. A multiple indicator composite of common indicators of network centrality/position and a ranking of individuals scoring in the top 10th percentile on all three indicators provided us with a more nuanced perspective on the placement of KBs within a network. Policymakers were defined as those who could influence policy within the national ministry. The policymaker networks of six SPHs in Kenya explored through the SNA resulted in an appreciation of the social capital of academic faculty and the identification of academic KBs and are reported elsewhere [48]. This SNA led us to identify seven KBs in four of the SPHs.

In order to explore the question on perceived human capital, namely the individual attributes, capacities and skills of academic KBs and how these affect their roles, we employed an exploratory approach for data collection through semi-structured interviews. From March to May 2013, we requested face-to-face interviews with all seven KBs and also all seven Deans/Directors of SPHs (one SPH had two directors) and 37 of the 109 policymakers identified in the SNA whose contact information was available. We expected a realistic response rate of 10–12%, similar to other studies [50, 51]. We also interviewed one additional faculty who, although part of the SNA, did not mention contacts by name and therefore was not able to be fully mapped or considered for their potential as a KB. However, we included this faculty member because of his large (unidentified) policy network, long history of involvement with policy decisions and position of respect by academic colleagues as an expert in health policy issues. We excluded faculty who did not appear to be directly engaged with policymakers.

Interviews were prefaced with “*There are many labels attached to people who serve as a bridge between two distinct communities. In this context we are interested in those who play a role in bridging the divide between research and policy. In so doing, these actors attempt to ensure that policies are evidence informed and simultaneously attempt to ensure that research is policy relevant. I would like to ask your opinion on some of the characteristics of such persons*.” Questions of interest covered (1) opinions on individual characteristics that facilitate knowledge brokering; (2) ranking of the above on importance; (3) opinions on personal characteristics that hinder knowledge brokering; (4) factors that hinder KBs from being effective (for KBs) and which of the previously mentioned barriers should be a priority for alleviation (for policymakers). Given the lack of consensus among researchers investigating this question as to the individual attributes likely to support KBs, our interview guide was not informed by a specific framework or theory.

The interview guide was pilot tested in country and modified accordingly. The study was approved and supported by the Dean of each SPH who facilitated communication with the faculty. Initial contact was made by email with up to two follow-ups via telephone and text messages.

Semi-structured interviews lasted between 30 and 90 minutes. Each interview was conducted in English, audio-recorded (with participant consent) and transcribed verbatim.

### Data analysis

Transcripts were reviewed against the original recordings for accuracy prior to coding. Given the dearth of comprehensive and contextually relevant frameworks, transcripts were coded with ATLAS.ti software using an open-ended approach employing inductive thematic analysis [52, 53]. This resulted in a detailed coding template with code categories corresponding to each major domain in the interview guide. Two members of the research team independently coded a subsample of transcripts to ensure validity and inter-rater reliability. Complete transcripts and codes were entered into ATLAS.ti to generate reports for each key domain. Nomenclature and categorisation were further refined during manuscript preparation.

For the purpose of this paper, the primary domain “KB skills and personality characteristics”, consisted of five item codes: expertise, networks, communication, soft skills, and political knowledge. Additionally, we consulted SPH and Government documents to verify respondent assertions where relevant, particularly in instances where there was uncertainty about institutional policies and practices. This served as a means of data triangulation.

## Results

The various attributes, capacities and skills of KBs that emerged from the study can be described under five categories: sociodemographics, professional competence, experiential knowledge, interactive skills and personal disposition. We describe these categories in Table 1 and the narrative below.

**Table 1:** Five categories of KB characteristics

Sociodemographics	Age
	Sex
	Academic qualifications
	Foreign training
Professional competence	Technical expertise
	Relevant research focus
	Leadership experience
Experiential knowledge	Practical experience
	Policy insight
Interactive skills	Creation and maintenance of networks
	Communication skills
	Interpersonal skills
Personal disposition	Social and moral conscience
	Determined and unrelenting
	Respectful
	Team player

### Sociodemographics

Twelve faculty were interviewed – five out of seven SNA-identified KBs and 10 faculty in leadership positions. Some individuals were identified as both leadership and KBs, so categories were not mutually exclusive. Four of the five KBs interviewed simultaneously held leadership positions. They were generally men with well-established academic careers (associate or full professors), who had been with their respective institutions for over 10 years. The one exception was a female senior lecturer. Of the six faculty in leadership positions who did not emerge as KBs, only three (50%) indicated being actively involved with policy decisions at the time of the study. No leadership from ESPUDEC were available for an interview.

Of 37 policymakers contacted, 13 agreed to be interviewed and 11 were ultimately completed. Respondents originated from four of the 16 national government agencies mentioned in a previous study [48], namely the Ministry of Public Health and Sanitation (MOPHS), Ministry of Medical Services (MOMS), Office of the President (PresOff) and Ministry of Livestock Development (MoLD). All four government agencies are responsible for formulation, implementation and monitoring of legislations, regulations and policies. While some conduct their own research, all indicate that they take into consideration evidence from a variety of sources to inform their decisions. All government respondents were senior national level policymakers comprising two ministry directors, two deputy directors, two division heads, two department heads, one chief and one deputy chief. Table 2 presents key characteristics of all respondents.

**Table 2:** Respondent overview

Sociodemographic characteristic	Knowledge brokers at the various SPHs^a^ (*n* = 5)	Leadership^b^ across the various SPHs (*n* = 10)	Policymakers across the various national ministries (*n* = 11)
Age (range in years)	47–67	51–67 (one outlier: 39)	50–73 (two outliers: 38 and 40)
No. of females	1	2	4
Highest level of education:			
Bachelor	–	–	–
Masters or MD	–	–	5
Doctoral (PhD)	5	10	5
Post-Doctoral			1
No. receiving tertiary degree from a foreign university (in addition to Kenya)	5	10	8
Academic position			
Lecturer	1	–	n/a
Senior Lecturer	1	5	
Associate Professor	2	3	
Professor	1	2	
Length of time at institution (range in years)	7–23 (median: 14 years)	3.5–38 (median: 17.5 years)	5–32 (median: 21 years)
No. who have worked in academia AND policy environment	5	7	4

^a^While not all institutions were classified as schools of public health (SPHs) within their organisations, we refer to them as SPHs in this study

^b^Categorisation not mutually exclusive (i.e. some members of SPH leadership were also KBs)

### Professional competence

#### Technical expertise

Several faculty indicated that their academic credentials, their subject specialisation and their technical expertise facilitated their roles as KBs. They noted that holding a PhD or equivalent degree was rare and therefore an asset in raising their profiles as experts. Training or experience in health policy was an added benefit as expressed here:

“… *so if you talk about doctors who have a background in say policy work. We are not more than five or six in the country so it’s not hard to find* [us]” (KB, SPHUoN)

Having received academic qualifications from renowned universities and serving as advisory or teaching experts overseas further enhanced faculty reputation.

#### Relevant research focus

Niche disciplines emerged as key for academics and policymakers alike. One faculty member stressed academics should,

“… *be focused in an area of expertise because then you become visible.…and therefore to be sought after when there’re issues that require policy engagement…regardless of who is in* [office]*, or what the political structure is like*” (Leadership, KB, GLUK-TICH)

The same respondent cautioned, however, that such expertise should not necessarily be gained from single isolated projects, but rather from a portfolio of work that is focused and directed. A KB from SPHUoN advised academic researchers to tailor their interests to government priorities, or shape those priorities based on one’s expertise. The respondent signalled against getting too “*wrapped up in one particular issue – because the issue really depends on what’s the issue of the day. And so it would be easy to be pigeon-holed…then it’s hard to re-invent yourself.*” He provided the example of the rising concern over non-communicable diseases, “*Cancer has become very high profile because the last two ministers* [of health] *both had cancer. So suddenly there is a lot of opportunity.*”

Understanding that policymakers face competing priorities and receive attention from multiple researchers were important realisations for some faculty. They felt that it was important to place an argument within a ‘systems perspective’. Policymakers expressed appreciation for such understanding, explaining that,

“…*just because the health policies are not addressing the issue that is your concern, doesn’t mean that they* [policymakers] *are doing nothing*” (Former Policymaker)

In sum, being anchored in research that is applied and relevant to policy and practice was an advantage.

#### Leadership experience

Almost all of the KBs had a history of leadership positions. This was important for policymakers, one of whom compiled numerous attributes of KBs in this one statement,

“*We had to get somebody who can lead this team, who is well known in the government circles, who knows politicians quite well, that is also quite conversant with the health situation in the country*” (Policymaker, PresOff)

Although several faculty believed that leadership positions offered more opportunities for visibility and engagement in EIDM, the importance of seniority was met with mixed opinions, with one respondent in particular who challenged junior faculty to overcome the stereotypes, believing that they can,

“… *make a contribution to the policy based on your experience even if you have not been a big person in society*” (Leadership, KEMU)

While this latter statement is encouraging, Kenyan policymakers often do rely heavily on senior positions as a proxy for expertise, as demonstrated by policymaker emphasis on ‘protocol’ and the role of ‘authoritative’ figures.

### Experiential knowledge

#### Practical experience

Participants described the importance of extra-academic experiences that enhance faculty credibility, visibility and confidence. Several policymakers appreciated academics who were not only experts in their field of inquiry but also familiar with the complexities of policy and/or program implementation. Some policymakers expressed frustration, however, with academics who they felt had no operational experience, stating that recommendations that emerge from such faculty were not often anchored in practical realities. For example, discussing the 2009 Kenyan H1N1 outbreak, one policymaker said,

“*We had many experts including people from the universities. But of course in an outbreak scenario, mostly they come to learn not really to* [give] *expert advice. They have theory* [but] *when we deal with an outbreak, which requires more practical input….We have other organisations which are very good like the WHO, UNICEF, CDC*” (Policymaker, MOPHS)

Urgent epidemiological health concerns were not the only areas in which policymakers preferred practical experience. Issues relevant to health systems and operational research were also raised. This requirement of practical experience likely explains why KBs were generally older and had a variety of professional experiences – some directly embedded within government.

#### Policy insight

Throughout the interviews it became apparent that without knowledge of the political environment, the policy cycle, decision-making roles, and relationships between actors, faculty engagement with policymakers would be suboptimal. More importantly, policymakers argued that academics familiar with the structure of government were more likely to be strategic about their networks and interactions. In addition, KBs indicated that teaching provided opportunities for them to translate policy-relevant knowledge to academia (and not only academic knowledge to policymakers), thereby supporting the bi-directionality of their relations and knowledge. Regardless of source of political knowledge, KBs, leadership and policymakers were unanimous in underscoring its importance when attempting to encourage use of research during policy decision-making.

### Interactive skills

#### Creation and maintenance of networks

Kenyan policymakers identify in-country experts in multiple ways [54]. The most commonly described strategy was through personal networks and contacts. Policymakers used the terms ‘visible’, ‘exposed’, ‘credible’ and ‘reputed’ as they described the importance of academic prominence within their networks.

Although faculty acknowledged the traditional path of publishing in peer-reviewed academic journals as one method of gaining recognition in a given field, they also stressed the importance of personal and professional networks. Several faculty credited their ability to engage with policymakers and academics alike to former collegial encounters, alumni friendships, student-teacher interactions and political family connections. One faculty member, for instance, revealed that his previous engagements with government, private institutions, the World Bank, and UN bodies as well as with presidents,

“… *with presidents – both Moi and later Kibaki who was then the Minister for Home Affairs.… gave me the platform and confidence when I moved to the university that, I knew where to get certain support or help.*” (Leadership, KB, MUSOPH)

The onus of building these relationships and networks seemed to rest on the shoulders of academe. Several policymakers indicated that academic researchers need to be proactive in reaching out to them and ‘activist’ in maintaining their interest and attention as demonstrated here:

“*I would advise them to be, how do I put it? To be aggressive or something. They should also be planning to engage us and find out what’s new… Instead of waiting for those kind of other forums*” (Policymaker, MOPHS)

However, faculty respondents indicated that, for those already overburdened with teaching and research responsibilities, this proves a formidable challenge.

Faculty indicated that, while some colleagues were generous with their introductions, others were less comfortable sharing contacts. One KB explained why:

“*There’s always a bit of politics because it is a consultancy you are competing with some groups, so depending on the size of the consultancy, either you are working together, or working in competition for a specific bit of work.*” (KB, SPHUoN)

While competition may be one reason for protecting relationships, another faculty respondent, while happy to share experiences with policy influence and KB attributes, refused to discuss the details of the persons in his network. The reasons given were concerns over breach of privacy as well as hesitancy to reveal contacts that had taken years of effort to build and nurture.

#### Communication skills

The ability to communicate convincingly and in a timely fashion was raised by all respondents. Each emphasised various aspects of effective communication, which we categorise here into audience, message, medium and messenger [55].

##### Audience

Knowing your audience – their knowledge, skills and interests – emerged as critical to framing messages for policymakers:

“*You have to present your policy in a way that I, who has never seen the inside of a hospital, whose children never get pneumonia* – *but I make decisions about funding and everything* – *can understand, internalise, and say: ‘This is the way to go.’ It will not work with: ‘This is statistically significant’*” (Policymaker, MOPHS)

The respondent further cautioned that, when recommendations are contentious, preparing for the opposition is critical. One KB recommended sharing results with as many stakeholders as possible in advance of finalising the policy brief and that consensus building was important due to health being an emotive subject – an astute assertion confirmed by a Policymaker in the President’s Office: “*What you realise is some policy making is not only technical. Sometimes even emotions influence policy.*”

##### Message

While all policymakers appreciated rigorous methodology, they suggested minimising descriptions of the research process and focusing on the justifications, implications and recommendations. The practical relevance of research results and the operational aspects of policy implementation were emphasised repeatedly. For instance, while researchers urged rapid roll-out of pneumococcal vaccine, implementation processes eluded them, partly because of an under-appreciation of the complexities of cold chain storage and transport:

“*If I am doing this* [vaccine] *policy, it goes beyond saying ‘You’re going to save X babies’ lives.’ It has to be: ‘How do we need to implement it?’ …. And the guy told me: ‘Expanding the cold chain* […] *means that every health facility in any remote health in this country….needs a fridge,…and syringes… and power!’ And we said: ‘No no no, we can’t have fridges this size for drugs. Remove the needles. You need to put* [the vaccines] *in antimalarial packs and move anything else that doesn’t need to be in a fridge, before we can transport that.’ And that meant a whole change in the manufacturing process. And that’s what it takes; the practical parts. Coz it’s one thing to get the vaccines to Nairobi, but it doesn’t mean a thing if a child in Lodwar cannot get it.*” (Policymaker, MOPHS)

In line with logistical concerns, the economic implications of policy recommendations were important. No faculty mentioned the cost-effectiveness of policy recommendations explicitly as part of their communication strategy, but some appeared to be aware of its importance. All KBs independently mentioned providing policymakers with solutions and not just problems. Furthermore, a member of leadership suggested including global evidence to support local arguments to enhance the importance and credibility of the research.

Policymakers further explained that communication messages needed to be succinct, relevant and easy to understand not because the content was necessarily above their intellectual thresholds, but because of time constraints and, more importantly, because they – the policymakers – would ultimately be the ones defending the policy options.

##### Medium

Printed mediums for research results such as policy briefs, short two- to three-page summaries, cabinet memoranda, and strategy papers were a popular request from all policymakers. A member of SPHUoN leadership supported condensing voluminous studies into succinct briefs by further noting that “*when you do scientific work, you have to…sieve it out for policymakers to be able to understand, because they don’t have time to read your treatises…*” With respect to oral presentations, policymakers and faculty indicated that messages should be short and impactful. What they would like to avoid is more experiences such as the following:

“*We had one lecturer from* [name of university redacted]*…He was given fifteen minutes; he lectured for one hour. He was boring people but he could not get that he was boring… After that meeting, we were forewarned by one of our seniors not to invite those kind of persons who are coming to waste peoples’ time…because they are not able to differentiate policy issues and academic issues*” (Policymaker, MOPHS)

In addition, the impact of graphics in presentations was highlighted:

“*If it is a line graph, a bar chart, if it’s a pie chart, it makes more sense to a policymaker than dry figures*” (Policymaker, MOMS)

While faculty involved in policy influence expressed the need to “demystify” research to engage both policymakers and the public, they expressed frustration that the packaging of research results for a policy audience was not deliberately taught through SPH curricula, and therefore such capacity was relegated to few individuals who had attended communication workshops or had gained the skills over time.

##### Messenger

While communication skills were considered essential for KBs, there were differing views on who should communicate research implications. Some policymakers acknowledged that,

“*Normally when somebody presents somebody else’s piece of work, there could be questions coming out of it and he’s not able to do it quite articulately. What we need to work on is, get* [the academic researchers] *to do the big pieces of work that they do. But also get them to know that they can present that big piece in fifteen minutes effectively that can actually bring the policy change. Because at the end of it, why are we doing all this research and all? To influence policy!*” (Policymaker, PresOff)

Others wondered if this specialised skill could be outsourced to specially trained people or leveraging these skills amongst existing faculty. A policymaker alluded to respect for authority and protocol in Kenya, indicating that it might be almost detrimental rather than advantageous for individual researchers to present their own results and recommendations:

“*We have the line of command …You need to have the professor or somebody at that level communicating to the director, so that is also very important. Because it bears more weight when it is communicated, for example, by the Principal Investigator in the project.*” (Policymaker, MoLD)

This may explain, to some degree, why six of the seven identified KBs had previously or currently been in a position of leadership at the SPH.

#### Interpersonal skills

While demonstrating network-building skills suggests the benefits of extroversion, social etiquette was considered essential regardless of one’s social predisposition. Leadership from several SPHs drew on past experiences to emphasise what they deemed simple, but important, behaviours, such as timely responses to policymaker emails, avoiding inappropriate or informal language, and wearing suitable attire for various occasions. Several other characteristics of academic KBs were charisma, humility, being accessible to policymakers, and possessing negotiation skills. While two policymakers raised ‘diplomacy’ as an important character trait, one described it as public relations while the other described it as a plea for understanding the various competing pressures of policymakers. However, in both cases, the essence of the assertion was the need for political and social astuteness.

### Personal disposition

#### Moral and social conscience

One key characteristic that resonated throughout the interviews was a deeply felt moral obligation to utilise research as a means to improve public health and save the lives of fellow citizens. Policymakers and faculty believed that, in order to be a KB, to go beyond traditional academic values of publications and teaching, faculty needed to be internally motivated and driven by a social conscience that would serve to propel action:

“*It’s not easy to change mindsets; It’s not easy to get your agenda heard; It’s not easy to come up with a communication strategy. But if you believe in what it is that you try to do, and you believe in getting people to see things in a different way, then you’ll be successful*” (Faculty member, KEMU)

One policymaker asserted that there would be a welcome change to see researchers going beyond their silos and publications to demonstrate the impact their work can have on society.

Several KBs and leadership emphasised that the only way to ensure that these passions materialise was to engage with policy as well as with the public.

“*If you want to transform lives of people in Kibera - the slum area there - you don’t go there with all theories that you know about sanitation;…you have to go and listen to them and engage them…and feel that they are part and parcel of you, even if you’re an academician, a professor, you must stoop low, and go and work with the people;… you have to sit with those people, listen to their stories it requires a lot of patience*.” (Policymaker, PresOff)

Time constraints, particularly for faculty bearing high teaching loads, were cited as barriers to knowledge brokering. However, for those who persevered, creating time was considered crucial. One policymaker in particular felt strongly about this:

“*I’ve seen senior professors….they think they have no time, but you’ll not have time to do anything than leave a mark!*” (Policymaker, PresOff)

Financial and logistical barriers also dominated KB deliberations: many respondents carefully weighed the immediate costs of time away from work, transport, parking, etc. versus the potential positive outcomes of influencing policy.

#### Determined and unrelenting

Proactivity and persistence emerged as necessary attributes of academic KBs not only in the context of networking but also in the context of research to policy. The majority of policymakers interviewed insisted that the onus of ensuring that research results were considered by government rested on the shoulders of the researchers:

“*we had to make sure that within that team we also have a good advocate. Somebody who’s not scared of speaking to policymakers*” (Policymaker, PresOff)

However, one policymaker cautioned that there is a delicate line between persistence and insistence:

“*I don’t know how to describe it but sometimes you have these pushy characters who think they can push their way into Government… Present the good results and wait for change to happen in its own good time…It is a process so you cannot afford to be pushy.…you can only listen*” (Policymaker, MoLD)

Crossing this line can lead to needless resistance which, as one policymaker from the PresOff asserted, can be overcome by patience.

Faculty were aware of the potential for proactive engagement to reap little reward. Nevertheless, the need to be proactive, despite the risks, was unanimous among KB respondents, several of whom provided examples of when they ‘pushed’ for their cause. Although they acknowledged being led down the slippery slope of ‘activism’ or ‘lobbying’, it ultimately allowed them to be heard and to make the difference they sought.

#### Respectful

Respect emerged as a constant thread throughout discussions on KB characteristics. It was embedded in positions of leadership as demonstrated above. Performance in one’s area of expertise also seemed to invite respect. For instance, policymakers who understood peer-review publications as the metric for academic performance indicated using this as one indicator of a faculty member’s respective expertise. In addition to being respected, being respectful was emphasised as equally valuable. Patronising, arrogant or derisive attitudes were frowned upon and likely to lead to disinterested policymakers:

“*I think that the most obnoxious behaviour from academics is to treat everybody as ignorant.* […] *There’s no substitute to a respectful approach. If you’re disrespectful…I think it really kills the goose before it even lays the eggs*” (Former Policymaker)

Policymakers in this study expressed frustration at being considered ‘less educated’, particularly because several held postgraduate qualifications. They therefore felt competent enough to not only understand the research but also interrogate it. Furthermore, mutual respect and trust were seen as necessary due to the interdependency of the two parties in satisfying their obligations to public health. Effective listening skills were also important. Some respondents, academics as well as policymakers, argued that being narrow-minded rendered academics blind to the complexities of the policy and practice arena whereas active listening and lateral thinking allowed for more receptive attitudes. A policymaker warned that without being open to ideas and suggestions from others, academics risked being relegated as “*ivory tower theorists*” with a “*citadel mentality*”.

## Discussion

We found considerable consensus in terms of the attributes, skills and capacities required of academic KBs in Kenyan SPHs, which we characterised into five overarching themes: sociodemographics, professional competence, experiential knowledge, interactive skills and personal disposition, recognising that there is substantial overlap and interconnection between these categories. We situate our findings within the broader literature before discussing the implications for organisational policies and practice, study limitations and future research needs.

### Links to broader KT literature

Most identified KBs had a history of leadership roles, suggesting academic positions (e.g. professorship) were less important than administrative positions (e.g. program director). Vogel and Kaghan [56] argue that the function of administrators “*increasingly involves active brokering with worlds outside the university so that universities can better compete in a global marketplace while they simultaneously build increasingly complex relations with governments*”. However, since half of faculty in positions of leadership were not classified as KBs but were engaged in policy endeavours, it appears that leadership positions alone are not sufficient for knowledge brokering. Similar to Lewis’s experience in Australia, decision-makers nurtured relations that were both position-specific [33], i.e. principal investigators, and individual-specific [57], i.e. renowned researchers.

The required sensitivity to political and social imperatives in the policymaking process is prevalent in the literature [34, 58–60] and reflected in our findings. Both policymakers and faculty indicated that policy insight and a respectful appreciation of the place of academia in policymaking facilitated appropriate, meaningful and timely engagement. Direct experience with policymaking provided faculty respondents with not only influential networks, but also a better appreciation of the complexities and nuances of navigating political structures and processes. In turn, this facilitated their development as true academic KBs. Our findings indicate, however, that while practical experience and leadership together provide a greater platform for KB opportunities, they alone are not sufficient. Less tangible characteristics such as patience, proactivity, persistence, humility, respect and diplomacy, amongst others, were also important and acknowledged in other studies [40, 61, 62]. The characteristics required to be a KB therefore cannot simply be taught. Consequently, for those who have the inclination and the passion, developing into a KB requires time – time to build credibility, time to hone one’s expertise, time to gain practical experience, and time to build a multitude of networks – which likely explain why the majority of the KBs were more advanced in their careers.

Relationships between KBs and some leadership of SPHs and government in Kenya have benefitted from personal networks and social capital built over time. Academic KBs in this study indicated multiple explanations for their expanded policymaker networks, including family history of involvement in politics, past positions of leadership in NGOs, involvement in consultancies, and previous government positions. These connections provided KBs with credibility, perceived power to influence and preferential political access [22, 57, 63–65]. It is not surprising, therefore, that KBs and policymakers alike indicated that, for some faculty who are ‘connected’, there was a tendency to hold policymaker relations close due to concerns of competition and a fear of betraying established trust. These academics, therefore, were likely to be gatekeepers rather than KBs [27, 42, 66, 67]. In addition to personalities, the proclivity to share or withhold contacts might be situation dependent and/or institutionally incentivised. Further research could explore this.

### Implications

EIDM is both a technical and social process [17]. Several changes in Kenya are likely to compel academics to engage with policymakers at an enhanced level of debate, deliberation and discussion in the future. These changes include devolution of health services and financing to counties, emphasising advanced education for civil servants as underscored in the new Kenyan constitution [68]; restructuring of the public service with the aim of optimised competency-based staffing; requirements by the Universities Act of 2012 for academia to “*support and contribute to the realisation of national economic and social development; and disseminate outcomes of the research conducted by the university*” [69], and a new DfID-funded initiative designed to optimise the use of research in decision-making for Health in Kenya [70]. The ‘two distinct communities’ [1, 17] paradigm may therefore fade, giving way to explicit acknowledgement of collaborative and interactive approaches to knowledge exchange [45], whereby academics will need to be equally knowledgeable about Kenyan health policy structures and processes.

KBs identified in this study demonstrated a unique interplay of sociodemographic attributes, professional competencies, experiential knowledge, interactive skills and personal disposition. While some are innate, others can be learned and appear to be advantageous when employed strategically and collectively. Furthermore, their personalities reflected those of an “*entrepreneurial outsider* [versus conforming and obedient insider]*…who thrives on advocacy and change* [versus stability]” [40]. This introduces consideration of intrinsic motivations that drive change and validate the required time investment. The most common drivers mentioned were being propelled by a moral conscience, retaining a deep sense of social justice, and possessing a passion for research as a means to improving health.

While these characteristics cannot be taught, they can be acknowledged, appreciated and encouraged. Tailoring SPH hiring practices to deliberately consider academic, practical, and personal qualities [45, 71] would encourage employment of such individuals. Furthermore, it would also recognise such individuals who already exist but whose contributions and worth are measured predominantly through traditional metrics of academic success such as publications. As McCormack et al. [4] assert, “*the change agent role does not seem to require a formal position or formal authority, with social influence and social interaction being key components of the role*”. By proactively and deliberately hiring faculty with KB-specific characteristics, SPHs could enhance their collective social capital (“*the advantage created by a person’s location in a structure of relationships*” [27]) and human capital (“*a person’s advantage in terms of personal attributes*” [28, 29]), capacities and skills that facilitate the creation and maintenance of relationships [72].

A ‘human capital’ audit of academic faculty could provide SPHs a sense of the existing capacities and gaps across the organisation and inform strategies to fill any gaps and leverage assets. SPHs would benefit from exploring ways to recognise, support and leverage KBs with the ultimate hope that it would raise the profile of the SPH as a whole. SPHs without KBs might consider their role in public health decision-making and how best to create bridges for dissemination of SPH research and activities. Furthermore, embedding communication training for the policy and practice worlds – the audience, the message, the medium and the messenger – would alert SPH students and other academic faculty to potential challenges when engaging with policymakers. Such capacity strengthening would need to be coupled with mentoring of junior faculty by more senior or experienced KB faculty.

While the factors mentioned are divided into distinct categories that enhance the roles of KBs, we note that there is a delicate balance and interplay of all the characteristics of a KB, thereby requiring different combinations of skills for different purposes. Accepting that it is unrealistic to expect all faculty to exemplify these characteristics, we encourage SPHs to consider the importance of faculty with some or many of these attributes, skills and capacities – either individually or as a collective – in furthering the relevance and goals of the SPHs.

### Limitations

This study focused on KBs’ attributes and therefore emphasised the perspectives of KBs and policymakers who engage with KBs. However, we did not interview non-KB faculty, and therefore we did not capture how they perceive KB attributes and why they seek-out KBs for advice. Nominations of relatively senior policymakers, unreliable government email addresses, limited access to publicly listed personal email addresses, and dependence on contact information provided by faculty respondents limited the number of policymakers we were able to interview. However, previous studies investigating policymaker perceptions have also yielded small numbers ranging from 12 for the whole African region [50], to an average of 14 policymakers per country across six countries [51]. While more interviews may have provided additional potential strategies for engagement, sample size in qualitative research is guided often by value of information collected for which we are confident that we reached saturation [73]. Furthermore, as most policymakers interviewed had advanced academic degrees, this may have influenced their appreciation of the value of research and researchers in general. Policymakers with less positive attitudes towards research may have been less likely to respond, therefore presenting a selection bias. To address this potential limitation, we probed policymakers on negative attitudes and experiences with academic faculty.

### Further research

Given that internal attributes of individuals are not divorced from the external contributors that affect effective evidence-to-policy brokering, understanding the greater political, regulatory and sociocultural context within which academic faculty in Kenyan SPHs operate is critical in understanding the reasons why certain perceptions and behaviours exist. Particular attention to gender differentials would be of additional interest. Further research could perhaps explore the organisational/environmental aspects that affect knowledge brokering activities of faculty as well as interactions between researchers and policymakers. Incentive and reward structures could shed additional insight on the role and recognition of KBs.

While a social conscience and feelings of moral obligation to the public were highlighted as key for propelling academic faculty to engage with policymakers and peers, it would be naïve to believe these were the only motivators for knowledge brokering. Structural, personal and strategic reasons for engagement are likely important. Further research on intrinsic and extrinsic motivators for KB engagement with policymakers, prevailing attitudes surrounding the value of research, and incentives for EIDM would provide deeper understanding of some of the preconditions for engagement. Additionally, these research foci would shed light on the ‘why’ of academic knowledge brokering in Kenya, thereby complementing research on ‘who does it?’ [48] and ‘how do they do it?’ [54].

## Conclusion

There is growing recognition of knowledge brokering as a role, as a profession and as a strategy to bridge the research and policy arenas. Though academic KBs exist in Kenya, they are relatively rare [48], and in this paper we demonstrate their unique sociodemographic attributes, professional competencies, experiential knowledge, interactive skills and personal disposition. By recognising existing KBs and supporting new KBs through strengthening tangible skills and recognising less tangible personal characteristics, SPHs can perhaps contribute more meaningfully to enhancing public health policies and ultimately, better health.
